# Impact of severity, duration, and etiology of hyperthyroidism on bone turnover markers and bone mineral density in men

**DOI:** 10.1186/1472-6823-11-15

**Published:** 2011-08-06

**Authors:** El Hadidy M El Hadidy, Mohamed Ghonaim, Soma Sh Abd El Gawad, Mohamed Abou El Atta

**Affiliations:** 1 Internal Medicine Department, Faculty of Medicine, Mansoura University, Egypt; 2 Clinical Pathology Department, Faculty of Medicine, Mansoura University, Egypt; 3 Diagnostic Radiology Department, Faculty of Medicine, Mansoura University, Egypt

**Keywords:** Bone mineral density, Bone formation markers, Bon resorption markers, Hyperthyroidism, Osteoporosis

## Abstract

**Background:**

Hyperthyroidism is accompanied by osteoporosis with higher incidence of fracture rates. The present work aimed to study bone status in hyperthyroidism and to elucidate the impact of severity, duration, and etiology of hyperthyroidism on biochemical markers of bone turnover and bone mineral density (BMD).

**Methods:**

Fifty-two male patients with hyperthyroidism, 31 with Graves' disease (GD) and 21 with toxic multinodular goiter (TNG), with an age ranging from 23 to 65 years were included, together with 25 healthy euthyroid men with matched age as a control group. In addition to full clinical examination, patients and controls were subjected to measurement of BMD using dual-energy X-ray absorptiometery scanning of the lower half of the left radius. Also, some biochemical markers of bone turnover were done for all patients and controls.

**Results:**

Biochemical markers of bone turnover: included serum bone specific alkaline phosphatase, osteocalcin, carboxy terminal telopeptide of type l collagen also, urinary deoxypyridinoline cross-links (DXP), urinary DXP/urinary creatinine ratio and urinary calcium/urinary creatinine ratio were significantly higher in patients with GD and TNG compared to controls (P < 0.01). However, there was non-significant difference in these parameters between GD and TNG patients (P > 0.05). BMD was significantly lower in GD and TNG compared to controls, but the Z-score of BMD at the lower half of the left radius in patients with GD (-1.7 ± 0.5 SD) was not significantly different from those with TNG (-1.6 ± 0.6 SD) (>0.05). There was significant positive correlation between free T3 and free T4 with biochemical markers of bone turnover, but negative correlation between TSH and those biochemical markers of bone turnover. The duration of the thyrotoxic state positively correlated with the assessed bone turnover markers, but it is negatively correlated with the Z-score of BMD in the studied hyperthyroid patients (r = -0.68, P < 0.0001).

**Conclusion:**

Men with hyperthyroidism have significant bone loss with higher biochemical markers of bone turnover. The severity and the duration of the thyrotoxic state are directly related to the derangement of biochemical markers of bone turnover and bone loss.

## Background

Thyroid hormones are necessary for normal skeletal growth, maturation, basic metabolism, and bone turnover [1]. Hyperthyroidism is accompanied by osteoporosis or osteopenia with increased rates of bone formation and bone resorption with predominance of resorption [2,3]. And higher incidence of fracture rates [4].

Bone densitometric studies revealed that bone mineral density (BMD) is decreased in all skeletal sites, including spine, femur, radius, and total body in patients with hyperthyroidism [5]. However, the deleterious effect of excess thyroid hormone on bone is reported to be more accentuated in areas consisting mainly of cortical bone like femoral neck and forearm [3,6]. On the other hand, Jodar et al (1997) reported that, there is also, a generalized reduction of BMD in the axial skeleton [7]. However, assessment of bone mass in patients with hyperthyroidism is recommended in cortical bones than trabecular bones [8].

Biochemical markers that reflect remodeling or turnover of bone can be measured in urine or blood including resorption markers and formation markers [9]. Resorption markers include tartrate resistant acid phosphatase, and products of bone breakdown as calcium and bone matrix degradation products like hydroxyproline, pyridinium cross-links, and telopeptides [10]. Bone formation markers include alkaline phosphatase enzyme, and three products of bone matrix synthesis which are osteocalcin, amino-and carboxy-terminal procollagen l extension peptide [11,12].

It is reported that bone resorption markers, as urinary pyridinoline and deoxypyridinoline are increased 7-8 times in hyperthyroidism more than age and sex matched controls [13]. On the other hand, serum osteocalcin and bone-specific alkaline phosphatase, reflecting bone formation, were increased to a less degree compared to the increase of urinary pyridinoline cross-links, suggesting the imbalance between bone formation and resorption with subsequent bone loss in hyperthyroidism [2,14].

There is paucity of data about the relation between the degree of hyperthyroidism, its duration, or its etiology and the resulting bone changes. The aim of the present study was to evaluate the bone mineral density and selected bone turnover markers in patients with hyperthyroidism, also to elucidate the impact of severity, duration and etiology of hyperthyroidism on these biochemical markers and bone mineral density.

## Methods

### Subjects

This work included 52 male patients with hyperthyroidism, receiving antithyroid agents during a period of 1-7 years, having normal FT3, FT4 and TSH levels for at least 6 months prior to the study, among those who were attending the Endocrine Out Patients Clinic of Mansoura University Hospitals, 31 with toxic Graves' disease (GD) and 21 with toxic multinodular goiter (TNG). Their age ranged from 23 to 65 years. A control group of 25 healthy men with matched age were included, their age ranged from 25 to 63 years.

Exclusion criteria included: women to nullify the effect of gender on the bone status, patients with co-morbidity (hypo-and hyper parathyroidism, vitamin D deficiency, Cushing's disease, diabetic nephropathy, inflammatory bowel disease, malabsorptive disease or renal diseases) or on medication (steroid, bisphosphonates, calcium or vitamin D) influencing bone turnover. Also patients with history of previous surgery or radiotherapy to the thyroid gland were excluded.

All participants provided written informed consent after receiving oral and written information concerning the study. The study protocol was approved by local ethical committee of the hospital. All patients and controls were subjected to: Full clinical examination, thyroid function tests (serum free T3, free T4 and TSH), radioactive isotope scanning and uptake of the thyroid gland with Technicium 99.

### Methods

In all patients and controls bone mineral density (BMD) was determined by dual X-ray absorptiometry (DEXA, Hologic QDR 1000 Analyzer) of the lower half of the left radius as the non-dominant hand; data were expressed as a real density in g/m^2 ^and as Z-score which is calculated by substracting the mean BMD of an age, ethnicity and sex matched reference population from the patients BMD and dividing the difference by the SD of the reference population.

Special biochemical assays included serum total calcium, phosphorus, total alkaline phosphatase, bone-specific alkaline phosphatase (B-ALP), osteocalcin (OC) and carboxy terminal telopeptide of type l collagen (β-CTx). Also, urinary calcium, urinary deoxypyridinoline (DXP) and urinary creatinine, were done for both patients and control.

Blood samples were drawn after an overnight fasting from patients and controls and sera were separated and divided into 2 plain tubes. Serum obtained from one of them was used for estimation of total calcium, phosphorus and total alkaline phosphatase using Roche Cobas Integra 400 plus analyzer, Germany[15,16]. The other tube was centrifuged at 1500 × g for 15 min then serum was stored frozen at -70°C until analysis of the following:

- Serum bone specific alkaline phosphatase (B-ALP) concentration was measured with a non-competitive enzyme immunoassay technique (Quidel Corporation, San Diego, CA, USA) [17].

- Serum free T3, free T4 and TSH levels were assayed by electrochemiluminescence immunoassay using Roche Elecsys 2010 immunoassay analyzer, Germany [18].

- Serum osteocalcin was analyzed using an immunoenzymatic assay for quantitative measurement of intact human osteocalcin (h-ost) using a kit supplied by Biosource Europe S.A, Belgium, according to the method of Power and Fortell (1991) [19].

- Serum carboxy terminal telopeptide of type l collagen (β-CTx) was assayed by electrochemiluminescence immunoassay using Roche Elecsys 2010 immunoassay analyzer, Germany [20].

At the same time, urine were collected for twenty four hours, urine volume measured then 10 ml was stored in Falcon tubes at -70°C until assay of urine calcium, creatinine and deoxypyridinoline.

- Urine deoxypyridinoline was analyzed by Immulite Pyrilinks-D chemiluminscent enzyme-labeled immunoassay using Immulite-1000 DPC, Los Angeles, according to the method of Reid et al (2004) [21]. Urinary deoxypyridinoline (DXP) was corrected for urinary creatinine and a ratio of DXP in nmol/l/urinary creatinine in mg/dl was calculated.

### Statistical Methods

Data were statistically analyzed using SPSS computerized package by Fisher's exact test to compare differences in rates and Student t-test for differences in parametric data. Significance of correlation and the relative contribution of each variable were assessed by Spearman Rank correlation test. P value < 0.05 was considered significant.

## Results

Table 1: Showed the clinical data (as age, body weight, hight, body mass index BMI and duration of thyrotoxic state) and the results for thyroid function test for all hyperthyroid patients (GD&TNG) and controls.

**Table 1:** Clinical characteristics and thyroid functions tests of patients with hyperthyroidism (GD and TNG) and controls.

*Parameters*	*GD (n = 31)*	*TNG (n = 21)*	*Controls (n = 25)*	*P1*	*P2*
***Age (years)***	43 ± 10	44 ± 8	42 ± 11	>0.05	>0.05
***Body weight (kg)***	69.5 ± 8.0	68.0 ± 12.0	86.0 ± 10.0	<0.05	<0.05
***Hight (cm)***	170 ± 10	173 ± 10	173 ± 13	>0.05	>0.05
***BMI (kg/m^2^)***	27.5 ± 3.1	27.8 ± 2.8	30.2 ± 2.9	>0.05	>0.05
***Duration of disease (years)***	2.9 ± 1.2	3.0 ± 1.5	-	-	-
***Serum TSH (uU/ml)***	0.89 ± 0.47	1.01 ± 0.36	3.31 ± 0.76	<0.01	<0.01
***Serum Free T3 (pmol/l)***	6.14 ± 2.32	5.62 ± 1.42	4.57 ± 1.43	<0.01	<0.05
***Serum Free T4 (pmol/l)***	20.8 ± 6.2	19.8 ± 5.7	17.9 ± 4.26	<0.05	<0.05

P1: Graves' diseases (GD) vs control

P2: Toxic multinodular goiter (TNG) vs control

Significant P < 0.05

Table 2: In patients with GD and TNG, there were significantly higher serum levels of B-ALP (P < 0.01), OC (P < 0.0001), β-CTx (P < 0.01) compared to controls. Also urinary calcium/urinary creatinine ratio, urinary DXP and urinary DXP/urinary creatinine ratio were significantly higher in GD and TNG patients compared to controls (P < 0.01). At the same time, BMD was significantly lower in patients with GD and TNG compared to controls. However, there were non-significant difference in patients with Graves' disease compared to those with toxic nodular goiter, as regard bone formation markers (serum B-ALP and osteocalcin), bone resorption markers (serum β-CTx, urinary calcium/urinary creatinine, urinary DXP and urinary DXP/urinary creatinine ratio) and BMD (P > 0.05).

**Table 2:** Markers of bone turnover and BMD in patients with Graves' disease and toxic multinodular goiter versus control.

*Parameters*	*GD* *(n = 31)*	*TNG* *(n = 21)*	*Controls* *(n = 25)*	*Significant*		
				
				*P1*	*P2*	*P3*
***Serum B-ALP (KAU/l)***	4.9 ± 3.6	4.3 ± 2.6	2.2 ± 0.8	<0.01	<0.01	>0.05

***Serum calcium (mg/dl)***	9.5 ± 1.0	9.8 ± 1.0	9.8 ± 0.5	>0.05	>0.05	>0.05

***Serum phosphorus (mg/dl)***	3.9 ± 0.6	4.0 ± 0.6	4.0 ± 0.7	>0.05	>0.05	>0.05

***Serum osteocalcin (ng/ml)***	12.9 ± 4.0	11.9 ± 4.1	6.6 ± 1.6	<0.0001	<0.0001	>0.05

***Serum β-CTx (ug/l)***	4.5 ± 0.8	4.1 ± 0.6	3.3 ± 0.6	<0.01	<0.01	>0.05

***Ur. calcium/urinary creatinine ***	0.54 ± 0.07	0.49 ± 0.05	0.36 ± 0.05	<0.001	<0.01	>0.05

***UDXP (nmol/l)***	68 ± 24	73 ± 22	49.8 ± 26	<0.01	<0.01	>0.05

***UDXP/urinary creatinine ***	12.6 ± 5.5	13.5 ± 5.0	6.3 ± 1.8	<0.001	<0.001	>0.05

***BMD at lower radius (g/cm^2^)***	0.780 ± 0.215	0.799 ± 0.232	0.952 ± 0.170	<0.01	<0.01	>0.05

Data: Mean ± SD

Significant P at < 0.05

**Figure 1:**
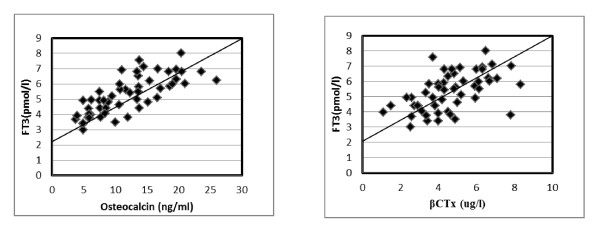
**Positive correlation of Serum FT3 with osteocalcin and β-CTx levels for all hyperthyroid patients**.

**Figure 2:**
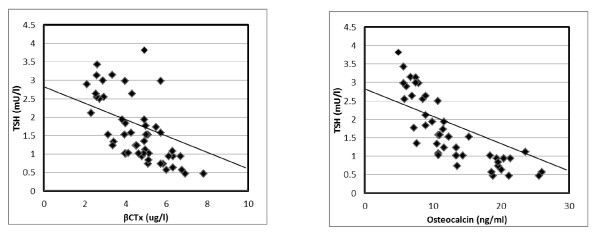
**Negative Correlation of serum TSH with osteocalcin and β-CTx levels for all hyperthyroid patients**.

Correlation between free T3 and biochemical markers of bone turnover revealed a significant positive correlation with all studied parameters. B-ALP (r = 0.37, P < 0.01), serum OC (r = 0.62, P < 0.001), serum β-CTx (r = 0.60, P < 0.001), urinary calcium/urinary creatinine ratio (r = 0.46, P < 0.01) and urinary DXP/urinary creatinine ratio (r = 0.52, P < 0.001). Also, correlation between free T4 and bone turnover markers revealed a significant positive correlation with B-ALP (r = 0.43, P < 0.01), serum OC (r = 0.65, P < 0.001), serum β-CTx (r = 0.65, P < 0.001), urinary calcium/urinary creatinine ratio (r = 0.61, P < 0.001) and urinary DXP/urinary creatinine ratio (r = 0.49, P < 0.01). While, significant negative correlation between TSH and biochemical markers of bone turnover was present, B-ALP (r = -0.39, P < 0.01), serum OC (r = -0.42, P < 0.01), serum β-CTx (r = -0.36, P < 0.01), urinary calcium/urinary creatinine (r = -0.26, P < 0.05) and urinary DXP/urinary creatinine ratio (r = -0.32, P < 0.05) (Figure 1&2).

Z-score of Bone mineral density measured at the lower half of the left radius in patients with Graves' disease (-1.7 ± 0.5) was not significantly different from those with toxic multinodular goiter (-1.6 ± 0.6) (table 3).

**Table 3:** Z-score of bone mineral density in Graves' disease patients, toxic multinodular goiter patients and controls.

	*Graves' Disease* *(n = 31)*	*Toxic Nodular* *goiter (n = 21)*	*Controls* *(n = 25)*	*Significance*		
				
				*P1*	*P2*	*P3*
***Mean ± SD***	-1.7 ± 0.5	-1.6 ± 0.6	-0.20 ± 0.26	<0.000111	<0.0001	>0.05

P1: Graves' disease vs controls

P2: Toxic multinodular goiter vs controls.

P3: GD vs TNG

**Figure 3:**
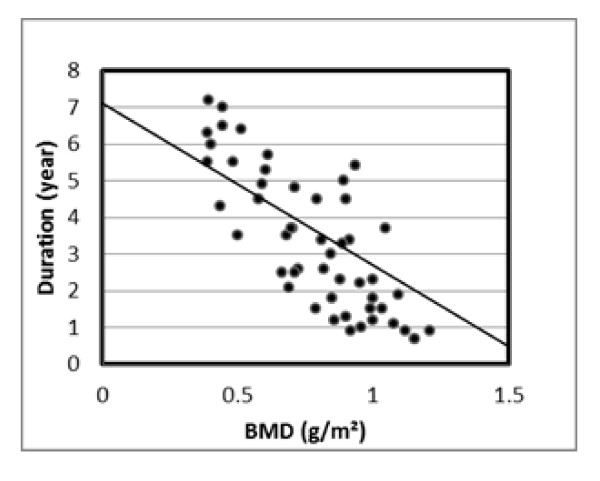
**Negative correlation of disease duration with BMD for all hyperthyroid patients**.

Correlation between the Z-score of BMD in hyperthyroid patients and free T3, free T4 as well as biochemical markers of bone turnover revealed a non-significant relation with any of them (r value for B-ALP -0.18, serum OC r = -0.14, Serum β-CTx r = -0.15, urinary calcium r = -0.09 and urinary DXP/urinary creatinine ratio r = -0.12) (table 4). While, correlation between duration of the thyrotoxic state and assessed bone turnover markers revealed a significant positive correlation with all of them. However, the BMD measured as real density (g/m^2^) or Z-score in the studied hyperthyroid patients was negatively correlated with the duration of thyrotoxic state (r = -0.68, P < 0.05) (table 5) (Figure 3).

**Table 4:** Correlation between Z-score of bone mineral density in hyperthyroid patients with thyroid hormones as well as markers of bone turnover.

*Free T3*	*Free T4*	*Serum* *B-ALP*	*Serum* *OC*	*Serum* *β-CTx*	Urinary calcium/ urinary creatinine	*Urinary DXP/* *urinary creatinine*
r = -0.12	r = -0.09	r = -0.18	r = -0.14	r = -0.15	r = -0.09	r = -0.12

P > 0.05	P > 0.05	P > 0.05	P > 0.05	P > 0.05	P > 0.05	P > 0.05

**Table 5:** Correlation between duration of thyroid disease with markers of bone turnover and Z-score of BMD at left radius.

	*Serum* *B-ALP*	*Serum* *Osteocalcin*	*Serum* *β-CTx*	*Urinary Calcium/* *urinary creatnine*	*UDXP/* *urinary creatinine*	*Z-score* *BMD*
***r value***	0.33	0.28	0.29	0.34	0.25	- 0.68

***significance***	<0.01	<0.05	< 0.05	< 0.01	< 0.05	< 0.001

## Discussion

Remodeling is essential for bone health. Remodeling is coupled with simultaneous formation and resorption of bone, but after middle age bone loss occurs because resorption exceeds formation. This imbalance is aggravated by many disease states [22]. Hyperthyroidism is one of the conditions associated with bone loss and increased fracture risk [4,23,24]. Biochemical markers that reflect remodeling can be assessed in blood or urine [9,13]. In the present work, we aimed to study some markers of bone turnover and bone mineral density and their relation to hyperthyroidism with different etiology.

The significantly higher bone formation markers (serum bone specific alkaline phosphatase and serum osteocalcin) and bone resorption markers (Serum β-CTx, urinary calcium and urinary deoxypyridinoline) in both patients with toxic Graves' disease and toxic multinodular goiter, found in the present work, confirms the high turnover state in the skeleton of patients with hyperthyroidism. There was a significant correlation between both free T3 and free T4 to B-ALP, serum OC as bone formations markers, and Serum β-CTx, urinary calcium/urinary creatinine ratio, urinary DXP and urinary DXP/urinary creatinine ratio as bone resorption markers. The high bone turnover markers in thyrotoxic patients with significant decrease in bone mineral density proved by the Z-score of the studied patients confirms the thyrotoxic osteopathy reported by many authors [2,4,9,14,23-26].

Thyroid hormones affect bone cells both in vitro and in vivo by stimulating osteoblast and osteoclast cells with more bone resorption and increased skeletal remodeling [27]. The exact mechanism of the deleterious effect of thyroid hormone on bone is poorly understood [23,24]. However, some authors reported that, bone loss in thyrotoxicosis may be as a direct stimulating effect of excess thyroid hormone acting locally on bone [4]. Also, TSH deficiency could be partly responsible for the skeletal loss seen in thyrotoxicosis [23], because it has been proposed that TSH may be a direct negative regulator of bone turnover acting via the TSH receptor on both osteoblasts and osteoclasts [24,28]. As TSH inhibits osteoclast formation and survival and also inhibits osteoblasts differentiation [29]. In addition, increased serum interleukin-6 concentration in hyperthyroid patients favors osteoclasts production may be effectors of the action of parathyroid hormones on bone [2,4].

Several factors may be incriminated in thyrotoxic bone loss [30]. Severity and duration of the hyperthyroid state are related to bone turnover and bone mass [11,31]. In the present study, duration of the disease was proved to be significantly related to bone mineral density as measured by real density and Z-score, also it was significantly related to the bone turnover markers, both of bone formation as serum B-ALP and serum OC, or bone resorption markers as Serum β-CTx, urinary calcium, urinary DXP and urinary DXP/urinary creatinine ratio. This comes in disagreement with Diamond et al (1994) who did not find a relation between the duration of the thyrotoxic state and the degree of bone loss in their study on pre- and postmenopausal women [32]. It is noteworthy that the present work studied men while, they studied pre-and postmenopausal women. Also, Nekrasova et al (2005) found that, the reduction in BMD most frequent and severe in patients with severe thyrotoxicosis but this loss of BMD does not depend on disease duration [31].

Bone densitometric studies proved that all sites of bone including axial and appendicular skeleton were affected in patients with hyperthyroidism [33], but Van de ven and Erdtsieck studies (2008) revealed that changes in cortical bone (appendicular skeleton like femur and radius) were more prominent than cancellous bone (axial skeleton as spine) [4]. In the present study, bone densitometry was done on the left radius and BMD Z-score for patients with toxic GD was not significantly different from that of patients with TNG. Furthermore, markers of bone turnover, whether of bone formation (serum B-ALP and OC) or bone resorption markers (Serum β-CTx, urinary DXP, urinary DXP/urinary creatinine ratio and urinary calcium/urinary creatinine ratio) were not significantly different in both categories of patients. This finding confirms the report of Jodar et al (1997) and Belaya et al (2007), they found no difference in bone mineral density in patients with Graves' disease compared to those with toxic multinodular goiter [7,34].

In the present work, although confirming decreased bone mineral density in the studied patients, there was a non-significant relation to either free T3 or free T4 or to any of the biochemical markers of bone turnover studied (B-ALP, serum OC, Serum β-CTx, urinary DXP cross-links, urinary DXP/urinary creatinine ratio and urinary calcium/urinary creatinine ratio). Variability in the duration of the thyrotoxic state could be the explanation behind the discordance between bone mineral density and severity of thyrotoxic state or bone turnover state.

## Conclusion

It is concluded that men with hyperthyroidism have significant bone loss with higher biochemical markers of bone turnover. The severity and duration of hyperthyroidism directly related to the derangement of biochemical markers of bone turnover and to the degree of bone loss. The etiology of the thyrotoxic state is not related to the degree of derangement in bone turnover markers or to the degree of bone loss.

## Authors' contributions

MG conceived of the study, participated in its design and coordination, sequence alignment and drafted the manuscript. SSA participated in the sequence alignment and drafted the manuscript and carried out the immunoassays and carried out the statistical analysis. EM participated in the design of the study and sequence alignment. MA participated in the design of the study and performed the bone mineral density. All authors read and approved the final manuscript.

## Pre-publication history

The pre-publication history for this paper can be accessed here:

http://www.biomedcentral.com/1472-6823/11/15/prepub
